# The Quantitative Trait Loci Mapping of Rice Plant and the Components of Its Extract Confirmed the Anti-Inflammatory and Platelet Aggregation Effects In Vitro and In Vivo

**DOI:** 10.3390/antiox10111691

**Published:** 2021-10-26

**Authors:** Jae-Ryoung Park, Rahmatullah Jan, Seul-Gi Park, Tri Handoyo, Gang-Seob Lee, Sopheap Yun, Yoon-Hee Jang, Xiao-Xuan Du, Taeho Lee, Yong-Sham Kwon, Doh Hoon Kim, Young-Mi Seok, Jong-Sup Bae, Kyung-Min Kim

**Affiliations:** 1 Division of Plant Biosciences, School of Applied Biosciences, College of Agriculture & Life Science, Kyungpook National University, 80 Dahak-ro, Buk-gu, Daegu 41566, Korea; icd92@knu.ac.kr (J.-R.P.); rahmat2021@knu.ac.kr (R.J.); uni@knu.ac.kr (Y.-H.J.); 2 Costal Agriculture Research Institute, Kyungpook National University, 80 Dahak-ro, Buk-gu, Daegu 41566, Korea; duxiaoxuan@korea.kr; 3 National Institute of Crop Science, Rural Development Administration, Jeonju 54874, Korea; ahsia1004@korea.com; 4 Department of Agronomy, Faculty of Agriculture, Jember University, Jl. Kalimantan 37, Jember 68121, Indonesia; trihandoyo.faperta@unej.ac; 5 Biosafety Division, National Academy of Agricultural Science, Rural Development Administration, Jeonju 54874, Korea; kangslee@korea.kr; 6 Graduate School of Science, Royal University of Phnom Penh, Sangkat Teuk Laak 1, Russian Federation Boulevard, Toul Kork, Phnom Penh 12101, Cambodia; yun.sopheap@rupp.edu.kr; 7 College of Pharmacy, Research Institute of Pharmaceutical Sciences, Kyungpook National University, 80 Dahak-ro, Buk-gu, Daegu 41566, Korea; tlee@knu.ac.kr (T.L.); baejs@knu.ac.kr (J.-S.B.); 8 Department of Genetic Engineering, College of Natural Resources and Life Science, Dong-A University, Busan 49315, Korea; kkmkim@hanmail.net (Y.-S.K.); dhkim@dau.ac.kr (D.H.K.); 9 Department of Korean Medicine Development, National Institute for Korean Medicine Development, 94, Hwarang-ro, Gyeongsan-si 38540, Gyeongsangbuk-do, Korea; imaria@nikom.or.kr

**Keywords:** cochlioquinone, defense, platelet aggregation, QTL, rice

## Abstract

Unpredictable climate change might cause serious lack of food in the world. Therefore, in the present world, it is urgent to prepare countermeasures to solve problems in terms of human survival. In this research, quantitative trait loci (QTLs) were analyzed when rice attacked by white backed planthopper (WBPH) were analyzed using 120 Cheongcheong/Nagdong double haploid lines. Moreover, from the detected QTLs, WBPH resistance-related genes were screened in large candidate genes. Among them, *OsCM*, a major gene in the synthesis of Cochlioquinone-9 (cq-9), was screened. *OsCM* has high homology with the sequence of chorismate mutase, and exists in various functional and structural forms in plants that produce aromatic amino acids. It also induces resistance to biotic stress through the synthesis of secondary metabolites in plants. The WBPH resistance was improved in rice overexpressed through map-based cloning of the WBPH resistance-related gene *OsCM*, which was finally detected by QTL mapping. In addition, cq-9 increased the survival rate of caecal ligation puncture (CLP)-surgery mice by 60%. Moreover, the aorta of rat treated with cq-9 was effective in vasodilation response and significantly reduced the aggregation of rat platelets induced by collagen treatment. A cq-9, which is strongly associated with resistance to WBPH in rice, is also associated with positive effect of CLP surgery mice survival rate, vasodilation, and significantly reduced rat platelet aggregation induced by collagen treatment. Therefore, cq-9 presents research possibilities as a substance in a new paradigm that can act on both Plant-Insect in response to the present unpredictable future.

## 1. Introduction

The Green Revolution of the 1960s led to a notable acceleration in grain production in developing countries, mainly due to the development of high-yielding hybrid strains of rice (*Oryza sativa* L.), wheat and maize, and the introduction of chemical fertilizers, pesticides and irrigation. However, because of their genetic homogeneity, the cultivars were more susceptible to pests, weeds and diseases than the traditional varieties. Approximately half of the world population uses rice as a staple food. Pests are the main culprits for the rice productivity reduction. White backed planthopper (WBPH; *Sogatella furcifera* Horváth) and brown planthopper (*Nilaparvata lugens* Stal, BPH) are among the major pests causing the greatest damage to rice crops worldwide. Both the pests transmitting southern rice black-streaked dwarf virus (SRBSDV), but WBPH is a strong persistent-transmitting vector for SRBSDV. In the near past, SRBSDV was discovered in Guangdong province China and rapidly spread in Southern China and Vietnam [1,2]. SRBSDV belongs to the Fijivirus genus which also includes *Oat Sterile Dwarf Virus* (OSDV), *Garlic Dwarf Virus* (GDV), *Fiji Disease Virus* (FDV), *Mal de Rio Cuarto virus* (MRCV), *Maize rough dwarf virus* (MRDV), *Pangola stunt virus* (PaSV) and *Nilaparvata lugens reovirus* (NLRV). These are all viruses that propagate in vivo through a hopper vector in a sophisticated way except GDV whose vector is still unknown [3].

A distinctive feature of plants and other sessile organisms, which cannot run away in case of hazard and lack an immune system to contest pathogens, is their capacity to produce a massive variety of compounds, the so-called secondary metabolites [4]. The biosynthesis of numerous secondary metabolites is constitutive, whereas in various plants it can be induced and enhanced by biological stress conditions, such as wounding or infection [5]. Over 2000 plant species are known to have pesticidal properties, and many of these plants are used by farmers in developing countries [6,7]. However, it is assessed that only 20–30% of higher plants have been studied so far [4]. Only a small percentage of plants has been screened for pesticidal activity, and, in addition, many such studies are not complete and often bioassay procedures used have been insufficient or inappropriate [8,9]. The plant kingdom represents a huge pool of new molecules to be discovered; as potentially useful biological compounds remain undiscovered, unexplored, undeveloped or underutilized from this reservoir of plant material [10,11]. To properly investigate the new compound, require separation techniques, structural elucidation and bioassay.

Because plants are sessile organisms that cannot move they face a variety of stress conditions during their growing life. Moreover, in order to survive this stress condition, numerous changes occurring in the growth stage and numerous mechanisms that can grow and develop by resisting stress have evolved [12]. Plant secondary metabolites are derivatives of primary metabolites made directly from plants due to a variety of physiological changes [13]. In addition, secondary metabolites play a key role in plant growth and survival under stress conditions and have a long-term effect [14]. In plants, about 100,000 secondary metabolites exist in three main groups [15]. In particular, plants synthesize aromatic amino acids such as tryptophan, phenylalanine and tyrosine, which are aromatic amino acids through the shikimate pathway. Aromatic amino acids are precursors to secondary metabolites that play a critical role in plant growth, development and defense reactions. In addition, chorismate, a precursor of aromatic amino acids, is used as a precursor of salicylic acid, a representative plant defense material.

QTL is a technique that is effectively used to analyze the relationship between phenotype and genotype [16]. So far, resistance genes and major QTLs that can be usefully used in agriculture have been reported [17]. However, these QTLs only use phenotype data, and studies that map using the concentrations of secondary metabolites and compounds that actually occur in cells are rare.

The accumulation of our novel compound is still unknown that whether it accumulated due to the viral (SRBSDV) infection or due to the WBPH wound. However, theoretically we can say that there is a close relationship between cq-9 and virus infection. Furthermore, cochlioquinone has leishmanicidal properties that can inhibit malaria-causing protozoan growth [18]. Due to the compound’s novelty and structural similarity, our study aimed at that along with WBPH resistance activity

Mostly, significant secondary metabolite synthesis in plants usually begins with the shikimic acid pathway, a complex metabolic pathway used by bacteria, fungi, algae, parasites and plants for the biosynthesis of aromatic amino acids. In animals and humans, this pathway is not found, so the essential aromatic amino acids must be obtained from plants or other organisms. The secondary metabolites are glycosides, alkaloids, carbohydrate, proteins, lipids, tannins, flavonoids, terpenoids, steroids, polyphenols, phytosterols, resins, glycoalkaloids, etc. and humans use them pharmaceutically. Human chronic diseases include cardiovascular diseases, diabetes, cancer and neurodegenerative and age-related diseases, and sepsis. Studies have suggested that isothiocyanates, catechin, quercetin, anthocyanidins, proanthocyanidins, lycopene, lutein and zeaxanthin are protective against various types of cancers [19]. Innovation of new drugs against these diseases is a crucial need, and natural sources such as plants with their tremendously diverse secondary metabolites may play an important role.

We have investigated a novel compound (cq-9) quite similar to cochlioquinone accumulated during WBPH stress condition through QTLs analysis. In this research, both phenotype data and secondary metabolite concentrations were used for QTL mapping; although it may be phenotypically resistant, but not in cell. The QTL mapping was analyzed using the concentration of resistant substances actually produced in cells, and the common region of QTLs using phenotype data and secondary metabolites was utilized. In addition, we analyzed whether the secondary metabolites produced by plants to resist viruses could be used in animals. It is still unknown whether the accumulation of our novel compound is due to viral (SRBSDV) infection or due to the WBPH wound. However, theoretically we can say that there is a close relationship between cq-9 and virus infection. On the basis of quinone and catechol being present in the cochlioquinone skeleton (similar in cq-9), it is predicted that it will be affective in treatment of cancer and sepsis diseases. Moreover, quinone greatly inhibits doxorubicin-resistant human breast cancer MCF-7/DOX cell proliferation and catechol inhibits lung cancer [20]. Furthermore, cochlioquinone has leishmanicidal properties and can also inhibit malarial causing protozoan growth [18]. Due to the compound novelty and structural similarity our study aimed that along with WBPH resistance activity, cochlioquinone could be possibly involved in anticancer and anti-sepsis activity.

## 2. Materials and Methods

### 2.1. Plant Materials

The Cheongcheong/Nagdong double haploid (CNDH) lines used for constructing the genetic map were obtained by in vitro anther culture of the F_1_ plants derived from crossing Cheongcheong (WBPH-resistant) and Nagdong (WBPH-susceptible) at Kyungpook National University. Cheongcheong is indica type rice cultivar with high yield and a complete abscission layer originating from *O. nivara*. Nagdong is a leading cultivar in the regional area with a partial abscission layer on the pedicel tissues and has been planted for over 20 years. The CNDH lines were cultivated in a paddy field 3 years after it was developed in 2010. For the anther culture, anthers were cultured through a two-step method [21]. In order to distinguish the resistance and susceptibility of the CNDH lines to WBPH, and 14 days after seeding, the WBPH was treated and the phenotypes were compared. When treated with WBPH, resistant lines showed green leaves, but susceptible lines had dried leaves. The resistance score was assigned to the CNDH lines through the comparison of phenotypes after treatment with WBPH. The phenotypes of the 120 CNDH lines were screened for WBPH resistance using the standard evaluation system of WBPH damage to rice.

### 2.2. WBPH and Rearing

The insectarium room was maintained at 27 ± 1 °C and 60–70% relative humidity with light illumination for 16 h/day. Thirty insects were placed in six bins every at preservation area until oviposition occurred [22], at which stage, they were transferred to 12 cages for selection of 2nd and 3rd instars. For breeding, the 2nd and 3rd instars WBPH were selected and transferred to the rice seedling, which had been prepared in mass plastic cages, to produce the next generation homogeneously. The WBPH could redistribute themselves onto the fresh plants. At 9–10 days post oviposition, the 1^st^ instar hatched from the egg, and after 14 days, the 2nd and 3rd instar nymphs were selected to infest the seedling stage.

### 2.3. QTL Analysis

The chromosomal locations of the QTL were resolved by composite interval mapping (CIM) of the genetic and bioinformatic data using Windows QTL Cartographer 2.5 [23]. We used a candidate gene map of the 120 CNDH lines with a set of resistance-related candidate gene markers (217 markers loci). The main window of Windows QTL Cartographer 2.5 lets in motion between open files, manipulate of evaluation parameters and display of chromosome graphics. Display parameters were set to show the LOD profile as a block sketch view, and the ratio between the effect on window measurement and LOD window size. QTL mapping was analyzed using the data collected from 2016 to 2020. First, we checked if the polymorphic markers in Cheongcheong and Nagdong were using 423 SSR markers for high-density mapping of the CNDH lines. Of these, 222 (52%) SSR markers were polymorphic. The total length of the related maps is 2121.7 cM and the average distance between SSR markers is 10.6 cM. The QTL was analyzed by the method of Composite Interval Mapping (CIM) of Win QTL cart 2.5, using the resistant score and concentration data and genotype information of the WBPH resistance substance in the CNDH lines.

### 2.4. Extraction of Compounds in Rice

A 500 g sample of the leaves was ground in liquid nitrogen, and then 500 mL of 70% methanol was added, and the mixture was shaken overnight at room temperature. The crude extract was filtered through filter paper. The pellet was washed with 500 mL of 70% methanol and shaken overnight at room temperature. Both supernatants were pooled into a separation tube and washed with hexane:chloroform (1:1 *v/v*) three times. The supernatant was collected into an evaporation tube and concentrated on a rotary evaporator. The residue (brown color) was blended with silica gel 60 (70–230 mesh), and 25 g of the silica gel was packed into a glass column (2.5 cm in diameter). The eluent was collected into glass tubes (5 mL/tube) and dried using a heating block at 50 °C.

### 2.5. Purity Assessment of cq-9 by Thin Layer Chromatography

In the methanol extract of rice, cq-9 was accurately separated by TLC (Thin Layer Chromatography). One microliter of methanol extract at 10 mg/mL was loaded on a TLC plate (60 F254 plates, Merck, KGaA, Darmstadt, Germany), and the developed solvent was mixed with chloroform, methanol, butanol and DW at a ratio of 4:5:6:2 was developed. The developed TLC plate confirmed the spot at 254 nm, which is a short wavelength, and 365 nm, which is a long wavelength of the UV lamp. In response to WBPH, we identified cq-9 as a new compound and were patent in USA under the patent number US 10,562,911_B2.

### 2.6. HPLC Determination of cq-9

For HPLC profiling, SMs were extracted from cells, as well as from liquid medium in which the cells were grown, using methanol. The cells were separated from the medium by centrifugation and then mixed with 20% methanol and sonicated, while the liquid fraction was mixed with 20% methanol without sonication. After centrifugation, the supernatant was collected and washed with an equal volume of n-butanol, which was then evaporated on a rotary evaporator. The TLC sample previously diluted in methanol was separated by reverse-phase HPLC into peak (retention time = 8 s), using a waters HPLC system (consisting of a 1525 pump, 2487 detector and 717 Plus autosampler), equipped with a Zorbax column (4.6 × 250 × 10 mm, particle size 5 mm; Agilent). Acetonitrile and 1% acetic acid were used as the mobile phase at a flow of 1 mL/min for 50 min. Detection was by UV at 250 nm.

### 2.7. Confirmation of Molecular Weight and Chemical Structure of cq-9 through LC/MS

We used LC/MS to analyze the materials, with an MSQ Plus Single Quadrupole Mass Spectrometer (Thermo Fisher Scientific, San Diego, CAWaltham, MA, USA). The infusion concentration was a 1:1000 sample dilution using 50% methanol in 0.1% formic acid and the flow rate was 50 µL/min.

### 2.8. Isolation of RNA and Construction of cDNA Library

Total RNA samples were isolated from leaves harvested 14 days after planting. The standard protocol and chemicals supplied with the QIAprep Spin Miniprep kit (QIAGEN, Cat. 27106, Hilden, Germany) were used. The cDNA was synthesized using a qPCRBio cDNA Synthesis kit (PCR Biosystems, Cat. PB30.11.10, Wayne, PA, USA) based on the manufacturer’s instructions. Specific primers (forward: 5’-ATGGCGGCGGCGATGATTCTCTCCTGCA-3’; reverse: 5’-TCAGGCATTGCAAGTTCGAATCCTA ACAAG-3’) for the ORF with *Bam*H1 and *XhO*1 restriction sites were designed for the PCR. PCR was performed with Pfu high-fidelity polymerase enzyme (Bioneer, Cat. K-2301, Daedeok, Daejeon, Korea) in a total volume of 50 µL. The following PCR conditions were used: initial denaturation at 94 °C for 5 min, followed by 40 cycles of denaturation at 94 °C for 30 s, annealing at 58 °C for 30 s, and extension at 72 °C for 1 min; and a final extension at 72 °C for 5 min. The amplicons were then purified from 1% agarose gel.

### 2.9. Bacteria, Yeast Strains and Media

The strains *E. coli* DH5α and *S. cerevisiae* D452-2 were used in this experiment. The yeast episomal plasmids pRS42k, PGK1p and CYC1t were used in both *E. coli* and *S. cerevisiae*. YPD medium (1% yeast extract, 2% peptone, 2% glucose) was used as the basal medium for the routine growth of yeast, as described previously [24]. After autoclaving and cooling the solid and liquid media until 45°C, geneticin (G418) and spectinomycin (Invitrogen, Cat. 11860038, Waltham, MA, USA) were used at 150 mg/L for selection [25].

### 2.10. Cloning and Vector Construction

The construct used for transforming the yeast was prepared in three steps: insert preparation, vector construction and ligation. To insert the amplified gene, as previously described, the insert was purified after gel electrophoresis using a QIAquick Gel Extraction kit (QIAGEN, Cat. 28706X4, Hilden, Germany). The purified insert (2 µg) was treated with *Bam*H1 (New England BioLabs, Cat. R3136S, Ipswich, MA, USA) and *Xho*1 (New England BioLabs, Cat. R0146S, Ipswich, MA, USA) restriction enzymes (2 µL each) with Cutsmart buffer (4 µL) (New England BioLabs, Cat. B7204S, Ipswich, MA, USA) and incubated at 37 °C for 4 h. To digest methylated DNA, 2 µL of *Dpn*1 enzyme was added to the restriction digest, which was then incubated at 37 °C for 2 h. Additionally, the pRSk42 vector (3 µg) was also treated with *Bam*H1 and *Xho*1 enzymes (2 µL each) by incubating at 37 °C for 4 h. After cutting with the restriction enzymes, the vector was then treated with CIP (New England BioLabs, CatM0525S, Ipswich, MA, USA) to dephosphorylate the ends of the vector. Finally, the insert was ligated to the vector at an insert:vector ratio of 5:1 in the presence of Quick Ligase and 2× Quick Ligase Reaction buffer (New England BioLabs, B6058S, Ipswich, MA, USA). This construct was then transformed and propagated in *E. coli* JM109 cells. The plasmid used in this experiment was pRS42k, which derives from the pRS series of yeast episomal plasmids and acts as a shuttle vector between yeast and *E. coli*. Usually, pRS42k is not used for gene expression as it has no promoter and terminator sequences. To construct this plasmid as the expression vector pRS42k-PGK1p, we performed the following steps: the PGK1 promoter and CYC1 terminator site, along with the *OsCM* gene (Accession No. XM_015793648.2), were inserted into the pRS42K plasmid, as presented in 5 kb with the appropriate restriction sites. The restriction enzymes *Kpn*1 and *Sac*1 were used for the insertion of the whole fragment (promoter + gene + terminator). This plasmid backbone is mostly used as an expression vector in yeast because it can be easily manipulated to introduce foreign DNA into yeast. It is an independent and high copy number replicating plasmid containing the 2µ circle cloned at one of the 2µ circle *Eco*RI sites [26,27]. The 2µ circle fragment allows for the efficient replication of the plasmid in yeast [26]. It has been reported that the 2µ circle is present in almost all strains of *S. cerevisiae* at 50–100 copies per haploid cell [28,29]. The replication of pRS42k in bacteria, as well as in yeast, eases the isolation of specific genes of interest. Here, we propagated our gene of interest in *E. coli* and then used yeast for functional expression experiments. Due to the presence of the 2µ circle, the pRS42k plasmid can be efficiently transformed into yeast by the lithium acetate/single-stranded carrier DNA/polyethylene glycol (PEG) method [29], with slight changes. The colonies were grown for 6 days until the growth rate decreased, thereby showing a dependency on the substrate [30]. Often, yeast transformants may be affected by the structural instability of the vector due to a large foreign gene, which could be a reason for its decreased replication rate. Furthermore, episomal vectors are known to be structurally unstable when they contain a large foreign gene [31].

### 2.11. Transformation to Yeast

*Saccharomyces cerevisiae* was used in this study as a host for the recombinant protein, and the transformation was carried out with the lithium acetate (LiAc)/single-stranded carrier DNA/PEG method [29]. The yeast strain was grown in 10 mL of YPD medium at 30 °C overnight and then shaken at 200 rpm in a 250 mL YPD culture flask. After 12–14 h of incubation, the titer of the culture was determined by adding 10 µL of cells into 1 mL of water in a spectrophotometer cuvette to be read at 600 nm. Then, 2.5 × 10^8^ cells were added to 50 mL of prewarmed YPD into a pre-warmed flask to give a titer of 5 × 10^6^. The flask was incubated at 30 °C and 200 rpm for about 4 h. The cells were harvested and washed in 30 mL of water twice before finally resuspending in 1 mL of water by vortex. At the same time, the single-stranded carrier DNA solution (salmon sperm DNA; Sigma Chemical Co., D-1626, Saint Louis, MO, USA) was incubated in a boiling water bath for 5 min for denaturation and chilled on ice immediately. Next, 50% (*w/v*) PEG and 1.0 M LiAC were prepared accordingly, and then 360 µL of the transformation mix (35 µL plasmid, 36 µL LiAC, 240 µL PEG and 50 µL of the single-stranded carrier DNA) was added to 100 µL of competent yeast cells and vigorously vortexed. The cells were incubated at 42 °C for 40 min in a water bath and then harvested by centrifugation for 30 s at full speed. The supernatant was removed, the cells were resuspended in 1 mL of distilled water, and 40 µL of the cell mixture was plated in each selection medium. The transformants were enumerated after incubating the plates at 30 °C for 3 days.

### 2.12. Plasmid Isolation and PCR Amplification

After confirming the transformation by colony PCR, the respective colonies were used to inoculate 5 mL of liquid YPD media for overnight growth at 30 °C and 200 rpm. RNA was isolated using the RNeasy Plant Mini kit (QIAGEN, Cat. 74904, Hilden, Germany). The concentration of RNA was quantified using a NanoDrop 2000 spectrophotometer (Thermo Scientific, ND-2000, Waltham, MA, USA). The cDNA library was synthesized with the qPCRBio cDNA Synthesis kit (SuperScript IV One-Step RT-PCR System, Thermo Fisher, Cat. 12594025, Waltham, MA, USA) using 3 µL of the RNA sample and 1 µL of the primer (100 pmol). A simple PCR was performed in a volume of 20 µL under the following conditions: 94 °C for 5 min; followed by 30 cycles at 94 °C for 30 s, 58 °C for 30 s and extension at 72 °C for 1 min; and final extension at 72 °C for 5 min. The amplicons were analyzed on a 1% agarose gel at 60 V for 50 min.

### 2.13. Protein Isolation and Western Blot Analysis

For Western blotting, the protein was isolated from the yeast strain, according to a previous method [32] with slight modifications. Ten milliliters of the yeast strain was collected in a 50 mL Falcon tube and centrifuged at 5000 rpm for 5 min at 4 °C. The supernatant was discarded, and the pellet was resuspended in 5 mL of TEK buffer solution (50 mM Tris at pH 7.5, 2 mM EDTA and 100 mM KCl) and centrifuged again at 5000 rpm for 5 min. The pellet was resuspended in 5 mL of TES buffer solution (50 mM Tris at pH 7.5, 2 mM EDTA, 0.8 M sorbitol, 20 mM β-mercaptoethanol and 2 mM phenylmethylsulphonyl fluoride) disrupted by bead beating. Next, 140 mM PEG3350 and 0.2 g/mL NaCl were added to the supernatant containing microsomes and immediately incubated on ice for 15 min. Afterwards, the sample was centrifuged at 10,000 rpm for 10 min, and the pellet was resuspended in 100 µL of TEG solution (50 mM Tris at pH 7.5, 2 mM EDTA and 40% glycerol). Protein concentrations were determined by the Bradford method (Bradford, M. M. 1976). Isolated protein in equal amounts (20 µg) was separated on a 12% polyacrylamide gel, as previously described by Laemmli (1970) [33]. After separation, the protein was electro-transferred to a nitrocellulose membrane and kept in blocking buffer (50 mM Tris-HCl at pH 7.4, 150 mM NaCl, 0.1% Tween 20 and 5% skim milk) for 90 min at room temperature, as described by Rippert et al. 2002 [34]. After washing with TBST (50 mM Tris-HCl at pH 7.4, 150 mM NaCl and 0.1% Tween 20) for 40 min, the membrane was incubated with corresponding primary antibodies at 1/1200 dilutions, and polyclonal goat anti-mouse IgG antibody (Invitrogen Cat. 31122, Waltham, MA, USA) was used as the secondary antibody at room temperature. Immunodetection was carried out by using ECL Western Blotting Detection Reagents (Amersham, Cat. RPN2235, Bundanggu, Seongnam, Korea) and an Image Quant™ LAS 4000 system (Gelifesciences, Cat. LAS 4000, Uppsala, Sweden).

### 2.14. Generation of OsCM Transgenic Rice

*OsCM*-overexpressing transgenic rice (OX-*OsCM*) plants were produced using Cheongcheong. Total RNA was isolated using the RNeasy Plant Mini Kit for cloning *OsCM*. The ORF (open reading frame) of *OsCM* (MH752192) was amplified using total RNA, and the amplified product was inserted into pENTR/D-TOPO (pENTR Directional TOPO cloning kit; Invitrogen) and then inserted into the gateway system (Gateway LR Clonase enzyme mix kit; Invitrogen) was inserted into the destination vector pSB11 for the expression of *OsCM*. The constructed vector was transferred to agrobacterium cells LBA4404 (Takara, Cat. 9115, Kusatsu, Shiga, Japan) by selecting the completely inserted *OsCM* through sequencing in *E. coli*. Constructed agrobacterium transformed into the callus of Cheongcheong. Cheongcheong seeds were sterilized for 10 min in 1% hypochlorite and then sterilized for 10 min with 70% ethanol. Then, it was washed with ddH_2_O and dried. The dried seeds were cultured for 2 weeks in N_6_ medium containing 2 mg of Auxin. In addition, induced callus was pre-cultured in N_6_ medium containing 2 mg of auxin for 3 days to increase vitality. Agrobacterium containing OX-*OsCM* vector was cultured at 28 °C for 3 days in YEP medium to transform into callus. Cultured agrobacterium was shacked with callus for 30 min. After the agrobacterium incubation, the culture was carried out in a co-culture medium in the dark for 3 days. Afterwards, the callus was washed with 500 mg/L carbenicillin, and after drying, cultured in N_6_ medium containing 50 mg/L hygromycin and auxin under light conditions (16/8 h photoperiod) for 2 weeks. After that, the callus that survived the selection medium was transferred to the regeneration medium containing NAA and kinetin. Plants that were shooting and rooting in the regeneration medium were transferred to soil after purification treatment.

### 2.15. Development of Transgenic Progenies and Field Experiments

In T_0_ generation of OX-*OsCM*, PCR and qPCR were analyzed for confirm the overexpression lines which are inserted with *OsCM* (data not shown), and T_1_ seeds were harvested for developed next generation. In the T_1_ generation, T_2_ seeds of OX-*OsCM* with stable gene expression were selected through molecular biological methods, and then breeding on a field for each spike, and T_3_ seeds were harvested in bulk. The planting distance was 30 × 15 cm. The amount of fertilization was N-P_2_O_5_-K_2_O=9-4.5-5.7 kg/10a, which was bred according to the Agricultural Science and Technology Research Research Standard of Rural Development Administration.

### 2.16. Animals for In Vivo Permeability Assay

All animal procedures were approved by the Animal Experimental Committee of National Institute for Korean Medicine Development (NIKOM) and carried out in accordance with the Guide for the Care and Use of Laboratory Animals (US National Institutes of Health Publications). Every effort was made to minimize both the number of animals used and their suffering. Seven-week-old male Sprague-Dawley (SD) rats were Orient Bio (Gyeonggi-do, South Korea). The rats were housed in a room under controlled conditions (23 ± 1 °C and 40–60% relative humidity) under a 12 h light/dark cycle with ad libitum access to water and standard laboratory diet. After 1 week of acclimatization, isometric tension measurement and blood sample collection were conducted.

### 2.17. Effects of cq-9 on Vascular Barrier Disruption under Septic Death Model

The cq-9 was artificially synthesized by confirming the structure and subsequently used for the sepsis experiment [33]. LPS (serotype: 0111:B4, L5293), Evans blue and crystal violet were purchased from Sigma (Cat. 0111:B4, L5293, Saint Louis, MO, USA). Human recombinant HMGB1 was purchased from Abnova (Cat. H00003146-AP41, Neihu District, Taipei, Taiwan). Fetal bovine serum and Vybrant DiD were purchased from Invitrogen (Cat. V22887, Waltham, MA, USA). Primary HUVECs were obtained from Cambrex Bio Science (Cat. 10HU-012, East Rutherford, NJ, USA) and maintained, as previously described [34,35]. HUVECs at passages 3–5 were used. Male C57BL/6 mice (6–7 weeks old, 27 g) were purchased from Orient Bio Co. (Cat. C57BL/6, Jungwongu, Seongnam, Korea) and acclimatized for 12 days before starting the experiment. Animals (five per polycarbonate cage) were housed under controlled temperature (20–25 °C) and relative humidity (40–45%), with a 12:12 h light:dark cycle. Animals received a normal rodent pellet diet and water ad libitum during acclimatization. All animals were treated in accordance with the Guidelines for the Care and Use of Laboratory Animals issued by Kyungpook National University and the study’s design was approved by the Animal Care Committee of the University (IRB No. KNU 2016-54). To induce sepsis, male mice were anesthetized with Zoletil^®^ (tiletamine and zolazepam, 1:1 mixture, 30 mg/kg) and Rompun^®^ (xylazine, 10 mg/kg). The CLP-induced sepsis model was prepared as previously described [36]. Briefly, a 2cm midline incision was made to expose the cecum and adjoining intestine. The cecum was then tightly ligated with a 3.0 silk suture 5.0 mm from the caecal tip and punctured once using a 22 gauge needle to induce high-grade sepsis [37]. The cecum was then gently squeezed to extrude a small amount of faces from the perforation site and returned to the peritoneal cavity. The laparotomy site was then sutured with 4.0 silk. In sham control animals, the caecum was exposed, but not ligated or punctured, and then returned to the abdominal cavity. Briefly, HUVECs were plated (5 × 104 cells/well) in Transwell plates with a pore size of 3 µm and a diameter of 12 mm and cultured for 3 days. Confluent monolayers of HUVECs were treated with LPS (100 ng/mL) for 4 h or HMGB1 (1 g/mL) for 16 h, followed by treatment with SFN. Transwell inserts were then washed with PBS (pH 7.4), and growth medium containing 0.5 mL Evans blue (0.67 mg/mL) and 4% BSA was added. Fresh growth medium was then added to the lower chamber, and the medium in the upper chamber was replaced with Evans blue/BSA. Ten minutes later, the optical density in the lower chamber was measured at 650 nm. For the spectrophotometric quantification of endothelial cell permeability in response to increasing concentrations of each compound, the flux of Evans blue-bound albumin across functional cell monolayers was measured using a modified two-compartment chamber model, as previously described [38,39].

### 2.18. Isometric Tension Measurement

Vascular tension was evaluated in thoracic aortic rings collected from SD rats. A vasoconstriction study was performed as described previously [40,41]. Thoracic aorta was excised and immersed in ice-cold, modified Krebs solution (in mM: NaCl 115, KCl 4.7, CaCl_2_ 2.5, MgCl_2_ 1.2, NaHCO_3_ 25, KH_2_PO_4_ 1.2, and dextrose 10). The aortas were cleaned of all connective tissue, soaked in Krebs-bicarbonate solution, and cut into four ring segments (3.5 mm in length). Some rings were denuded of endothelium by gently rubbing the internal surface with a forcep edge. Each aortic ring was suspended in a water-jacketed organ bath (6 mL) maintained at 37 °C and aerated with a mixture of 95% O_2_ and 5% CO_2_. Each ring was connected to an isometric force transducer (Danish Myo Technology, Skejbyparken, Aarhus N, Denmark). Rings were stretched to an optimal resting tension of 2.0 g, which was maintained throughout the experiment. Each ring was equilibrated in the organ bath solution for 90 min before the experiment measuring the contractile response after the addition of 50 mM KCl. To determine the effect of cq-9 on the maintenance of vascular tension in rat endothelium-intact or endothelium-denuded aortic rings, vascular contractions were induced using the thromboxane A2 agonist, U46619 (30 nM, 20 min). When each contraction reached a plateau, rice extract and cq-9 were added cumulatively (0.1–0.5 mg/mL) to elicit vascular relaxation. In the second experiment, we investigated the inhibition of the relaxation response by treating endothelium-intact aortic rings with Apamin (500 nM) and tetraethylammonium (TEA, 5 nM) NG-nitro-L-arginine methyl ester (L-NAME, 100 μM) for 30 min. After U46619 treatment, cq-9 was cumulatively added to the aortic rings.

### 2.19. Blood Sample Collection and Platelet Aggregation Assay

For the method used for blood sample collection and platelet aggregation refer to the work in [42]. The blood samples were collected up to the mark in sky blue capped vacutainer containing trisodium citrate, thus assuring 1:9 ratio of blood is to anticoagulant. The platelet-rich plasma (PRP) and platelet-poor plasma (PPP) were prepared using Tyroid buffer (137 mM NaCl, 2 mM KCl, 12 mM NaHCO_3_, 5.5 mM glucose, 1 mM MgCl_2_, 0.3 mM NaHPO_4_, pH 7.4) and centrifuged (2000 rpm, 7 min). Platelet aggregation was measured in a two channel aggregometer (Chrono-log Lumi-Aggregometer model 560-Ca, Havertown, PA, USA) at 37 °C with stirring (1000 rpm). Washed platelets were pre-incubated with various concentrations of either rice extract and cq-9 for 1 min in the presence of 1 mM calcium chloride (CaCl_2_), followed by stimulation with various agonists (Collagen, ADP, or thrombin) for 6 min with continuous stirring at 37 °C.

### 2.20. Statistical Analysis

Data were analyzed by the Student’s *t*-test using IBM SPSS version 21.0. Microsoft Excel 2013 was used to design the graphics and tables.

## 3. Results

### 3.1. Applied to QTL Mapping of Phenotype and Secondary Metabolites

In order to distinguish the resistance and susceptibility of the CNDH lines to WBPH, 14 days after seeding, the WBPH was treated and the phenotypes were compared. When treated with WBPH, resistant plants showed green leaves, but susceptible lines revealed dried leaves. The resistance score was assigned to the CNDH lines through the comparison of phenotypes after treatment with WBPH. In addition, HPLC analysis was performed to compare the secondary metabolites produced in the resistant and susceptible lines after inoculation with WBPH. The CNDH lines showed resistance and susceptible when inoculated with WBPH, and as a result of HPLC analysis, peaks appeared different at specific times and showed various concentrations (Supplementary Materials Figure S1). The concentration of the substance displayed when treating the CNDH lines of WBPH shows a continuous distribution showing a normal distribution, and it means a quantitative trait expression regulated by one or more genes. QTL mapping was performed using the data collected from 2016 to 2020. First, check the marker polymorphic in Cheongcheong and Nagdong were performed using 423 SSR markers for high-density mapping of the CNDH lines. Of these, 218 (52%) SSR markers are polymorphic. Of the 218 SSR markers, 198 SSR markers were used except that the positions on the chromosome were overlap or unknown, and a related map was created. The total length of the related maps is 2121 cM and the average distance between SSR markers is 10.6 cM.

**Figure 1:**
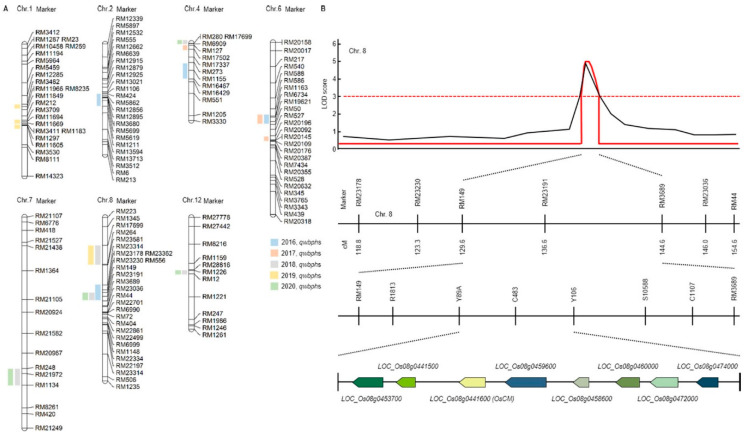
Quantitative trait loci (QTL) analysis and physical mapping related to white backed planthopper (WBPH). (**A**) RM23191 on chromosome 8 was commonly detected in 2016, 2018 and 2020. Eight candidate genes were detected for the WBPH resistance. (**B**) QTL analysis and physical mapping related to WBPH. When QTL mapping analyzed, the RM149-RM3689 region was detected with an LOD score of 3.0 or higher. When closed mapping is performed around this region, eight candidate genes (*Os08g0453700*, *Os08g0441500*, *Os08g0441600*, *Os08g0459600*, *Os08g0458600*, *Os08g0460000*, *Os08g0472000*, *Os08g0474000*) for the WBPH resistance were detected.

It is the result of QTL related to resistance to WBPH for 5 years in the CNDH lines (Figure 1A and Supplementary Materials Table S1). In 2016, qwbph2, qwbph4, qwbph6, qwbph7 and qwbph8 were detected. The qwbph2 is detected in RM5619-RM424 on chromosome 2, with a LOD value of 2.5 and an explainable phenotypic change of 10%. The qwbph4 is detected by RM273-RM16467 on chromosome 4 with a LOD value of 4.7 and an explainable phenotypic change of 18%. The qwbph6 is detected by RM50-RM1163 on chromosome 6 with a LOD value of 6.1 and an accountable phenotypic change of 29%. The qwbph7 is detected by RM21972-RM6776 on chromosome 7, with a LOD value of 4.8 and an explainable phenotypic change of 18%. The qwbph8 was detected by RM3689-RM23314 on chromosome 8 with a LOD value of 5.2 and an explainable phenotypic change of 18%. In 2017, qwbph4-1, qwbph6-1 and qwbph6-2 were detected. The qwbph4-1 is detected by RM127-RM17502 on chromosome 4 with a LOD value of 2.6 and an explainable phenotypic change of 16%. The qwbph6-1 is detected by RM50-RM1163 on chromosome 6 with a LOD value of 4.3 and an explainable phenotypic change of 23%. The qwbph6-2 is detected by RM20196-RM20096 on chromosome 6 with a LOD value of 2.7 and an explainable phenotypic change of 11%. In 2018, qwbph4-2, qwbph7-1, qwbph8-1, qwbph8-2 and qwbph12 were detected. The qwbph4-2 is detected by RM280-RM6909 on chromosome 4 with a LOD value of 3.5 and an explainable phenotypic change of 30%. The qwbph7-1 is detected by RM248-RM1134 on chromosome 7 with a LOD value of 3.0 and an explainable phenotypic change of 30%. The qwbph8-1 is detected by RM23230-RM3689 on chromosome 8 with a LOD value of 2.5 and an explainable phenotypic change of 30%. The qwbph8-2 is detected by RM17699-RM264 on chromosome 8 with a LOD value of 3.3 and an explainable phenotypic change of 30%. The qwbph12 was detected to RM1226-RM12 on chromosome 12, with LOD value of 2.7 and 40% phenotypic change explained. In 2019, qwbph1, qwbph1-1, qwbph1-2 and qwbph8-3 were detected. The qwbph1 is detected by RM3482-RM11966 on chromosome 1 with a LOD value of 4.0 and an explainable phenotypic change of 30%. The qwbph1-1 is detected by RM3709-RM11694 on chromosome 1 with a LOD of 3.5 and a 30% explainable phenotypic change. The qwbph1-2 is detected by RM11694-M11669 on chromosome 1 with a LOD value of 3.3 and an explainable phenotypic change of 30%. The qwbph8-3 is detected by RM17699-RM264 on chromosome 8 with a LOD value of 3.3 and an explainable phenotypic change of 30%. In 2020, qwbph4-3, qwbph6-3, qwbph8-4, and qwbph12-1 were detected. The qwbph4-3 is detected by RM280-RM6909 on chromosome 4 with a LOD value of 3.5 and an explainable phenotypic change of 30%. The qwbph6-3 is detected by RM248-RM1134 on chromosome 6 with a LOD value of 3.0 and an explainable phenotypic change of 30%. The qwbph8-4 is detected by RM23230-RM3689 on chromosome 8 with a LOD value of 2.5 and a 30% explainable phenotypic change. The qwbph12-1 is detected by RM1226-RM12 on chromosome 12 with LOD value of 2.7 and 40% phenotypic change explainable.

Finally, in the 5 years of QTL mapping results, Chromosomal 8 RM23191 was commonly detected in 2016, 2018 and 2020. In 2016 and 2018, the WBPH resistance score was used for QTL mapping, and in 2020, the concentration of substances generated through HPLP after WBPH inoculation was used for QTL mapping (Figure 1B and Supplementary Materials Table S2). Therefore, the result of using two different characteristics related to WBPH resistance was used to screening for candidate genes related to WBPH resistance by mapping markers in the same region among two different characteristics. For fine mapping, we used R1813, Y89A, C483, Y106, S10588, C1107 near RM23191. As a result, eight candidate genes (*Os08g0453700*, *Os08g0441500*, *Os08g0441600*, *Os08g0459600*, *Os08g0458600*, *Os08g0460000*, *Os08g0472000*, *Os08g0474000*) for the WBPH resistance were detected. *Os08g0453700* responds to plant defense responses from *Arabidopsis thaliana* to respiratory burst oxidase protein. *Os08g0441500* functions as a cinnamoyl-CoA reductase and responds to infection of pathogenic bacteria. *Os08g0441600* (*OsCM*) has a sequence similar to that of chorismate mutase CM2 and is involved in prephenate synthesis. *Os08g0459600* is an oxophytodienoate reductase and is involved in jasmonic acid biosynthesis. *Os08g0458600* is a signal transduction response regulator like Response regulator 33 or A-type response regulator 19. *Os08g0460000* is a Germin-like protein 1 precursor and is involved in plant defense. *Os08g0472000* is a transcription factor in abscisic acid-regulated transcription and is involved in plant defense and development. *Os08g0474000* is an AP2 domain-containing protein, a plant defense regulator.

### 3.2. cq-9 Extraction and Chemical Structure Analysis

**Figure 2:**
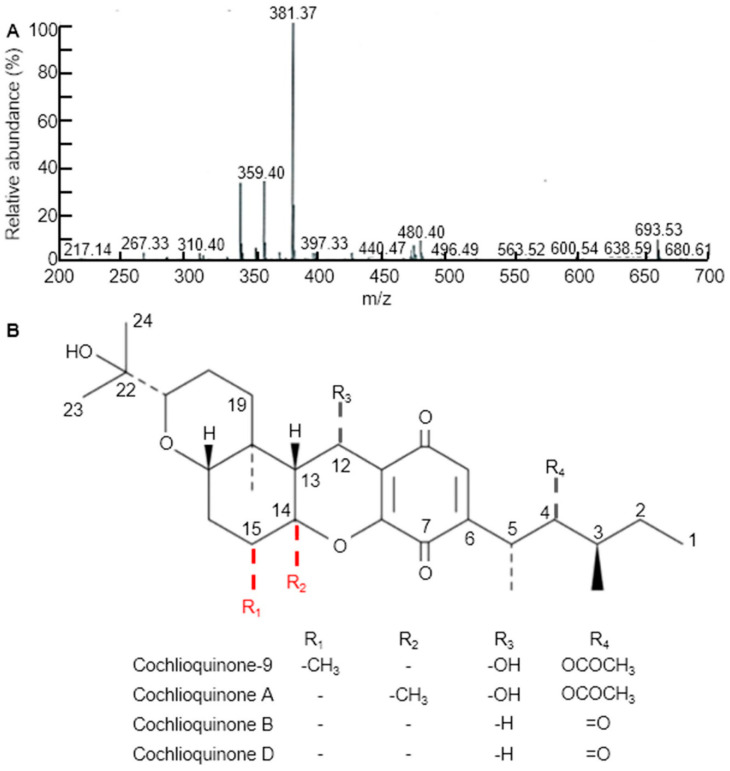
Extraction of substances related to Cochlioquinone-9 (cq-9). (**A**) Molecular weight of cq-9. Mass: 381.38. (**B**) Chemical structure of cq-9; (3R)-9-[(1S,2R,3S)-2-acetyloxy-1,3-dimethylpentyl] 1,2,3,4a,5,6,6a,12,12a,12b-decahydro-12-hydroxy-3-(1-hydroxy-1-methylethyl)-6a,12b-dimethylpyrano[3,2-a]xanthene-8,11-dione, C_30_H_44_O_8_, molecular weight = 532.7 g/mol.

Three weeks after WBPH infestation, infected leaves of all the examined rice cultivars and CNDH lines were sampling for extraction. Various solvent leaf extracts were obtained, dried, placed in separate falcon tubes and then completely immersed in a 50 mL solution of 90% methanol for 3 days in the dark. The relative concentrations of the metabolites were analyzed using TLC by comparing the mobility of the unknown compounds with spots of pure compounds used as control samples. The fractions of each extract containing the highest concentration of target compounds were pooled and evaporatively concentrated at 60°C. We further purified the metabolites by methanol extraction of the TLC silica, followed by high-performance liquid chromatography (HPLC), to determine the relative importance of cq-9 in rice. Comparative HPLC analysis of the control, Cheongcheong, Nagdong, TN1 and CNDH lines revealed amounts of cq-9 (data not shown). LC/MS analyses were conducted to identify the compounds. The LC/MS chromatograms of cq-9 shown in Figure 2A. Positive and negative LC/MS data revealed the molecular weight to be 381.38 and 480.40 *m/z* in cq-9. The chemical structural of cq-9 was identified (Figure 2B). The cq-9 which is similar to cochlioquinone having a little conformational difference.

### 3.3. OsCM Overexpression Plant Construct and Phenotype Evaluation

**Figure 3:**
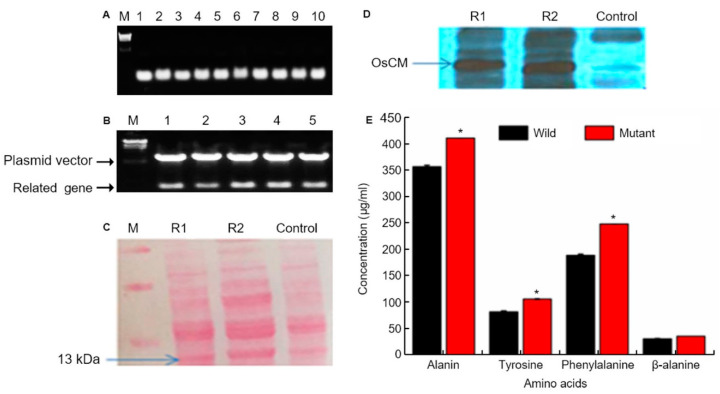
Chorismate mutase (CM) molecular analysis. (**A**) Colony PCR of 10 selected colonies of transformed yeast on the 0.8% agarose gel. And target gene size is 378bp. Lane; numbers 1 to 10 represent the selected colony number. M; λ/*Hin*dⅢ DNA ladder. (**B**) Confirmation of cloning and transformation of the gene of interest in *Escherichia coli* through restriction enzyme cutting. The upper band represents the vector, whereas the lower band shows the target gene size (378 bp). (**C**) Western blot confirmation of CM expression in yeast. Similar amounts of protein (20 µg) isolated from transformed yeast strain and control strain after 60 h. Following 12% SDS-PAGE, the gels were stained with Coomassie blue and treated with an antibody for the recombinant protein. (**D**) R1 and R2 represent OsCM replicate 1 and 2, respectively, which is the transformed strain, and the control is the wild type strain. The recombinant protein of the transformed strain is significantly expressed as compared with the wild type. (**E**) Alanine, tyrosine, phenylalanine and alanine biochemical analysis. The wild type is a control strain, and the mutant is transformed with the CM gene. * Significantly different at the 0.05 level.

We conducted colony PCR of 10 selected colonies of transformed yeast and checked the transformation (Figure 3A). We the ascertained confirmation of cloning and transformation of the gene of interest in *Escherichia coli* through restriction enzyme cutting. Colony PCR was used to select five colonies whose transformation was confirmed, and PCR was performed using *OsCM* primer (Figure 3B). As a result of PCR, transformation was confirmed in all five colonies. Western blot confirmation of *OsCM* expression in yeast. Similar amounts of protein (20 µg) isolated from transformed yeast strain and control strain after 60 h. Following 12% SDS-PAGE, the gels were stained with Coomassie blue and treated with an antibody for the recombinant protein (Figure 3C). The 13kDa band was thickened in the yeast transformed by *OsCM*. The recombinant protein of the transformed strain is significantly expressed as compared with the wild type (Figure 3D). Furthermore, analysis of major secondary metabolites. Alanine, tyrosine, phenylalanine and alanine biochemical analysis. The wild type is a control, and the mutant is transformed with the *OsCM*. Alanine, tyrosine, phenylalanine and β-alanine were all highly expressed in *OsCM*-transformed yeast. In particular, we analyzed the OsCM functional expression in yeast and were reported the highest quantity of Phe (249 µg/mL) and Tyr (108 µg/mL) (Figure 3E). Supplementary data (Supplementary Figure S1) online describes the role of CM in shikimate pathway and correlation of shikimate pathway with cq-9. In this pathway, the key step is the conversion of chorismate into prephenate, which is the main precursor of aromatic amino acids. Chorismate mutase enzyme is involved in the conversion of chorismate into prephenate. Phenylalanine is the precursor of the phenylpropanoid pathway, which is the intermediate of a high diversity of metabolites, including flavonoids and anthocyanins. The shikimate pathway produces salicylic acid (SA) in two ways; the iso-chorismate synthase enzyme converts anthraquinone into SA in the initial steps, while, in the last steps, phenylalanine undergoes a series of reactions and produces SA. SA is the ultimate source of catechol goes under various reaction, which is a key unit of cq-9.

**Figure 4:**
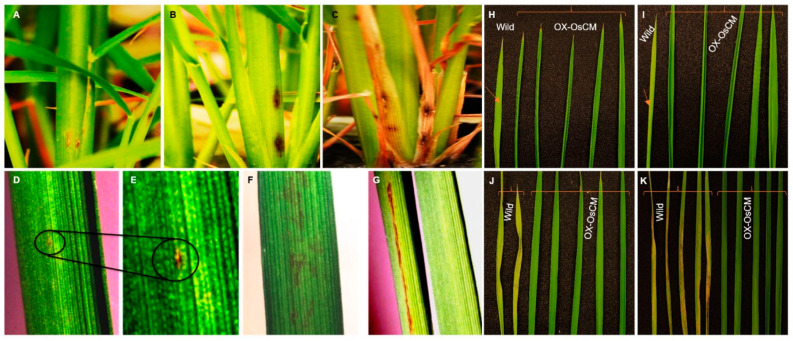
Phenotypic evaluation of transgenic and wild type rice under WBPH stress. Panel (**A**) represents OX-*OsCM*, a minor symptom that appeared after 30 days of infestation. (**B**,**C**) Cheongcheong and infection appeared very early, but the pictures taken after 20 days and 30 days, respectively, indicate that the infection increased very rapidly and finally the leaves died. (**D**,**E**) The spreading rate of infection in OX-*OsCM* leaf indicated with circles. (**F**,**G**) The pattern of spreading symptoms in Cheongcheong leaves. (**H**–**K**) The infection spreading rate after each time point and wild and OX-*OsCM* leaves differentiated with braces. Arrows in panels (H,I) indicate the initial infection site in Cheongcheong. Wild; Cheongcheong.

When WBPH was inoculated with OX-*OsCM* and Cheongcheong, various phenotypes were analyzed. In OX-*OsCM*, lesions rarely form and appear very small in size, but in Cheongcheong, many lesions form and persist for a long period of time (Figure 4A–C). Especially, Cheongcheong had a long lesion length, but OX-*OsCM* showed little lesion (Figure 4D–F). Moreover, Cheongcheong formed many lesions and died as the leaves turned brown and dry. However, OX-*OsCM* was a vivid green leaf, and the lesion was not largely formed (Figure 4G–K).

### 3.4. Anti-Septic Activities of cq-9

**Figure 5:**
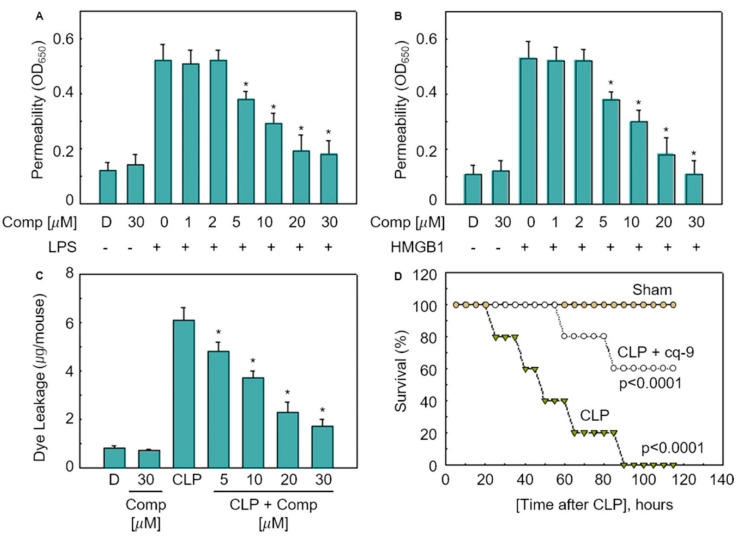
Effects of cq-9 on vascular barrier disruption under septic conditions and septic death model. (**A**) Effects of treatment with different concentrations of cq-9 for 6 h on barrier disruption caused by lipopolysaccharide (LPS), 100 ng/mL, 4 h. (**B**) High mobility group protein 1 (HMBG1), 1 μg/mL, 16 h were monitored by measuring the flux of Evans blue-bound albumin across human umbilical vein endothelial cells (HUVECs). (**C**) Effects of SFN on caecal ligation puncture (CLP)-induced vascular permeability in mice were examined by measuring the amount of Evans blue in peritoneal washings (expressed ug/mouse, n = 5). (**D**) Male C57BL/6 mice (n = 20) were administered compounds cq-9 at 12 and 50 h after CLP. Animal survival was monitored every 12 h after CLP for 132 h. Control CLP mice and sham-operated mice were administered sterile saline (n = 20). Kaplan–Meier survival analysis was used to determine the overall survival rates vs. CLP-treated mice. However, our human pathogenicity experiments did not show any effect. * Significantly different at the 0.05 level.

To study the permeability of the cq-9 compounds in vitro, human umbilical vein endothelial cells (HUVECs) were activated with lipopolysaccharide (LPS, 100 ng/mL) or high mobility group protein 1 (HMGB1, 1 mg/mL), followed by treatment with various concentrations of cq-9 for 6 h. Additionally, we also assessed the in vivo permeability via the administration of cq-9 into caecal ligation puncture (CLP)-surgery mice. We found that cq-9 dose-dependently inhibited LPS- and HMGB1-mediated membrane disruption (Figure 5A,B) or the peritoneal dye leakage, induced by CLP (Figure 5C). To determine whether cq-9 protected mice from CLP-induced sepsis lethality, we administered both compounds to mice after CLP. A single administration of cq-9 (12 h after CLP) did not prevent CLP-induced death (data not shown). Therefore, we next administered the same amounts of cq-9, but, this time, twice (once 12 h after CLP and once 50 h after CLP). The survival rate increased to 60%, according to the Kaplan–Meier survival analysis (*p* < 0.00001, Figure 5D).

### 3.5. The Role of cq-9 in Vasorelaxation

**Figure 6:**
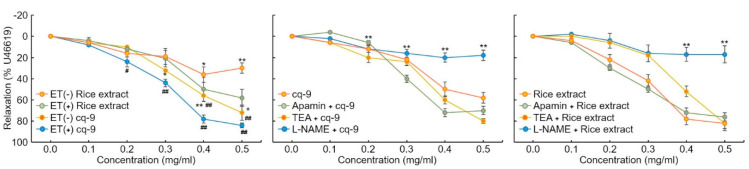
Rice extract or cq-9 reduces vascular contraction in SD rats. Vasorelaxation induced by rice extract or cq-9 in the aorta from rats. Rice extract or cq-9 was added cumulatively to elicit relaxation when vascular contractions induced by U46619 reached a plateau in endothelium-intact (ET+) or -denuded (ET-) rat aortic rings. We investigated the relaxation response by treating endothelium-intact aortic rings with Apamin (500 nM), tetraethylammonium (TEA, 5 nM) and NG-nitro-L-arginine methylester (L-NAME, 100 μM) for 30 min. After U46619 treatment, Rice extract or cq-9 was cumulatively added to the aortic rings. Relaxation is expressed as the percentage of the maximal contraction. * *p*<0.05, ** *p*<0.01 vs. Rice extract (ET-) or cq-9 (ET-). ^#^ *p*< 0.05, ^##^ *p*< 0.01 rice extract vs. cq-9. Data are expressed as the mean ± SE (n = 4).

We investigated the vasorelaxation response in aortic rings. We found that 30 nM U46619 caused contraction in aortic rings. Rice extract and cq-9 treatment induced vasorelaxation of both endo the lium-intact or endothelium-denuded aortic rings (Figure 6*)*. Here, rice extract or cq-9 treatment significantly increased vasorelaxation of endothelium-intact aortic rings than endothelium-denuded aortic rings. Furthermore, cq-9 treated aorta showed higher vasorelaxation compared to rice extract treated aorta. To further confirm the vascular relaxation effect of K^+^ channel blockers on rice extract or cq-9 induced relaxation, we pretreated with apamin, a blocker of Ca^2+^ dependent K^+^ channels, or tetraethylammonium (TEA), voltage-sensitive K^+^ channel (Kv) inhibitor, for 30 min in endothelium-intact aortic rings. Pretreated apamin (10 nM) and TEA (10 nM) did not alter aorta relaxation of rice extract or cq-9 (Figure 6). To determine whether rice extract or cq-9 relaxes vascular contraction through activation of the nitric oxide/cGMP pathway, we pretreated endothelium-intact aortic rings with NG-nitro-L-arginine methylester (L-NAME), a nitric oxide synthase blocker, for 30 min. Rice extract or cq-9 was added cumulatively to elicit relaxation when vascular contraction induced by U46619 (30 nM) reached a plateau. However, pretreatment with L-NAME resulted in significantly inhibited vascular relaxation induced by cumulative addition of rice extract or cq-9 (Figure 6).

**Figure 7:**
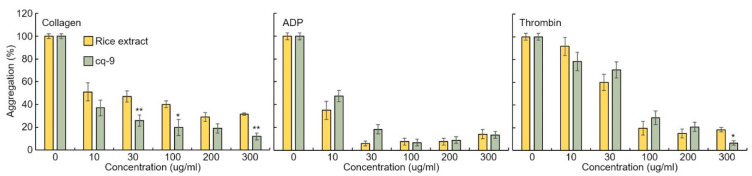
Rice extract or cq-9 inhibits agonist-induced rat platelet aggregation. Collagen-, ADP-, or thrombin-stimulated washed platelets pre-treated with Rice extract or cq-9 extract concentrations in the presence of 1 mM CaCl_2_. Platelet aggregation is expressed as the percentage of the maximal platelet aggregation by Collagen (0.625 μg/mL), ADP (5 μM), or thrombin (0.8 Unit). * *p*< 0.05, ** *p*< 0.01 Rice extract or cq-9. Data are expressed as the mean ± SE (n = 4).

Rice extract or cq-9 inhibit agonist-induced rat platelet aggregation. Rice extract or cq-9 inhibited rat platelet aggregation induced in a dose-dependent manner (from 300 μg/mL to 10 μg/mL) by several agonists, including collagen, ADP and thrombin (Figure 7). We also found that cq-9 extract significantly reduced rat platelet aggregation induced by collagen treatment compared to rice extract (30, 100, 300 μg/mL; *p* < 0.01, *p* < 0.05, *p* < 0.01).

## 4. Discussion

Development of the resistance rice and study on the defense genes with determination of natural products and breeding for pest-resistant plants is a predominant focal point of agricultural research. Agricultural traditions have made necessary contributions to our knowledge and shaped our perceptions on plant resistance to pests for the duration of plant molecular breeding. Particularly, rice resistance to WBPH is well studied in the context of defense genes and natural management of these pests in agriculture. The most efficient and economical way of controlling WBPH is by developing resistance rice cultivars, resistant genes and natural products. The genetic alteration of secondary metabolites pathways during stress condition produces side products which are most significantly involved in mitigation of human disease. QTLs analysis is a basic tool to determine the gene and pathway involved in the specific stress inducing tool. In our study, we analyzed the genes and pathways involved in WBPH stress through QTLs analysis. We detected QTLs associated with cq-9 of the CNDH lines putatively on chromosomes 1, 2, 4, 6, 7, 8 and 12, respectively. The physical map of each chromosome was completed using a sequence database (http://www.gramene.org/, accessed on 11 October 2020). The vertical lines in the physical map indicate the advent of complete genetic linkage map consisting of codominant DNA marker typically SSR markers. QTL associated with rice resistance were identified the genes on the regions of map base genetic contribution.

In the results, the additive effects of the QTL were positive with the additive coming fours QTL detected on chromosomes 1, 2, 4, 6, 7, 8 and 12, respectively. Regarding the additive effects, contribution of phenotype variation and genotype variation, a total of 21 QTLs identified by Windows QTL Cartographer 2.5, with one QTL (RM23191) detected on the same location. QTL mapping analyzes the interaction between genotype and phenotype and detects the sequence region most related to the phenotype [43]. In this research, we focused on the regions that are mapped identically every year through repeated experiments for 4 years.

In this research, the newly discovered gene *OsCM* was located on chromosome 8, and chromosome 8 showed a higher density than other chromosomes. Therefore, more accurate QTL could be obtained. From 2016 to 2019, there are 17 QTLs detected that show LOD of 2.5 or more when using the WBPH resistance score. Using the results of HPLC analysis, QTL mapping was carried out in 2020, and four QTLs were detected. RM23191 of chromosomes 8 was commonly detected in 2016 and 2018 when QTL mapping was performed using WBPH resistant score, and also detected in 2020 when QTL mapping was performed using the results of HPLC analysis after WBPH inoculation, centering around this region, we searched for candidate genes for WBPH resistance. In this study, we performed QTL mapping of WBPH resistant gene through WBPH resistant score and HPLC analysis for 5 years, and searched candidate genes centered on RM23191 of commonly detected chromosome 8. Here, eight candidate genes were detected (*OsNox6*, *OsCCR*, *OsCM*, *OsOPR7*, *OsRR33*, *OsGLP1*, *OsbZIP66*, *OsDERF3*).

In our research, we evaluated *OsCM* (accession number MH752192) as a candidate gene which induces in response to WBPH resistant. *OsCM* is a key enzyme in the shikimate pathway, which catalyzes a major step of converting chorismate into prephenate, a precursor of Phe and Tyr. The shikimate pathway provides aromatic compounds in prokaryotes, ascomycete fungi, apicomplexans and higher plants, but it is absent in mammals, which makes it an antibiotic target [44]. The conversion of chorismate to prephenate is a sigmatropic shift reaction in the shikimate pathway [34,45], which can also speed up the synthesis of a wide range of secondary metabolites through the synthesis of phenylalanine (Ph) and tyrosine (Tyr) [46]. Chorismate possesses a significant position in the shikimate pathway as it represents a main controlling linkage between primary and SMs synthesis in higher plants, which is controlled by *CM*. Furthermore, chorismate synthesized salicylate through isochorismate synthase [47,48], which is further involved in the synthesis of phylloquinone [49,50]. Cochlioquinone also known as luteoleersin [51] and the scientific name of basic skeleton is 17-methoxycochlioquinone proposed by Geris et al. 2009 [51]. It is a highly complex compound, and its biosynthesis is also very complex but here we will explain it briefly. Basically, this compound is composed of two basic units: one is a farnesyl group, which is also identical to oxadecalin, and the second unit is an acetogenin derivative of an aromatic compound, and we synthesized cochlioquinone by cycloaddition of both the segments. Moreover, we established the mixed biosynthesis of cochlioquinone through the introduction of a farnesyl unit onto an aromatic precursor whose secondary metal groups are provided by methionine which is produces in methionine biosynthesis pathway [52]. The first unit of cochlioquinone is farnesyl pyrophosphate which is synthesized from tyrosine in the shikimate pathway. Tyrosine is the ultimate source of acetyl-CoA synthesis by a series of reactions like oxidative decarboxylation of pyruvate and tyrosine like other amino acids (phenylalanine, tryptophan and lysine) catabolism. Furthermore, acetyl-CoA goes under a series of reactions in isoprenoid pathway (mevalonate kinase pathway) and gives isopentenyl pyrophosphate (IPP) which further pair with another molecule of IPP and produce geranyl pyrophosphate (GPP). This GPP is the accepter of another isoprene molecule and as a result produce farnesyl pyrophosphate (FPP) which is the precursor of all type of sesquiterpenes [53]. Here, it is important to mention that tyrosine and phenylalanine synthesis highly regulates by *CM*. The prenylation of acetogenin aromatic-derived nucleus leading to an intermediate complex and the decarboxylation–hydroxylation reaction converts it into another intermediate which is further converts to cochlioquinone compound by the cyclization of the farnesyl chain [52]. This prenyl accepter primarily produced by polyketides but also it can be derived from tyrosine which is an evidence of *CM* involvement in cochlioquinone synthesis [54]. Moreover, quinone is the basic moiety of this compound which is also a product of the shikimate pathway.

Chorismate mutase and shikimate pathway are also directly involved in another epi-cochlioquinone compound which is identical to cochlioquinone. In order to explain epi-cochlioquinone very easily it can be divided into two segments. The first one is catechol (1,2-dihydroxybenzene) and the second is oxadecalin, but *CM* is only concerned with the synthesis of first segment. The main skeleton of cochlioquinone would be synthesized by the cycloaddition of these both segments described by Hosokawa et al. 2010 [55]. Catechol would be synthesis by stereoselective manner of treatment of aminophenol with quinone which is the product of shikimate pathway. The reaction for synthesis of catechol is presumed that, first the aminophenol should be oxidized to iminoquinone which further gives o-quinone by receiving acid hydrolysis which works as an oxidant for aminophenol to synthesize catechol and iminoquinone through autoredox catalysis. And also phenylalanine is the precursor of benzoic acid which is first hydroxylated to SA at the ortho-position and then converted to catechol, moreover it is predicted that SA is directly converted to catechol due to oxidative decarboxylation [56].

Supplementary Material (Supplementary Materials Figure S2) describes the role of *OsCM* in the shikimate pathway and the correlation of the shikimate pathway with cq-9, which is also known as luteoleersin [51]. The scientific name of its underlying structure is 17-methoxycochlioquinone [57]. It is a highly complex compound, and its biosynthesis is also very complex. Cochlioquinone is composed of two basic units, one is a farnesyl group or also identical to oxadecalin, and the second unit is acetogenin derivative of aromatic compound and synthesise cq-9 by cycloaddition of both the segments [55]. The structure of cq-9 was compared with other structure of Cochlioquinone such as Cochlioquinone A, B and D. The difference between Cochlioquinone A and Cochlioquionone B is that -OH and -H are bonded to C-12 (R3), and -OCOCH3 and =O are bonded to C-4 (R4), respectively [58]. Cochlioquionone D is similar to B, but has a double bond of C-2 and C-3 [59]. The molecular structural of cq-9 is most similar to that of Cochlioquinone A, except that the methyl group is bonded to C-15 (R1) rather than C-14 (R2). The mixed biosynthesis of cq-9 occurs through the introduction of a farnesyl unit onto an aromatic precursor, whose secondary metal groups are provided by methionine, which is produced in the methionine biosynthesis pathway [52]. The first unit of cq-9 is farnesyl pyrophosphate, which is synthesized from Tyr in the shikimate pathway. Tyr is the ultimate source of acetyl-CoA synthesis, produced by a series of reactions, like oxidative decarboxylation of pyruvate and Tyr, and the catabolism of other amino acids (Phe, Trp and lysine). Furthermore, acetyl-CoA undergoes a series of reactions in the isoprenoid pathway (mevalonate kinase pathway) and gelites isopentenyl pyrophosphate, which further pairs with other isopentenyl pyrophosphate molecules and produces geranyl pyrophosphate. This geranyl pyrophosphate is the accepter of another isoprene molecule and, as a result, produces farnesyl pyrophosphate, which is the precursor of all types of sesquiterpenes [52]. Tyr and Phe synthesis is highly regulated by the *OsCM*. The prenylation of the acetogenin aromatic-derived nucleus, leading to an intermediate complex and the decarboxylation–hydroxylation reaction, converts it into another intermediate, which is further converted to cq-9 by the cyclisation of the farnesyl chain [52]. This prenyl accepter is primarily produced by polyketides, but it can also be derived from Tyr, which is evidence of the involvement of *OsCM* in cochlioquinone synthesis. Moreover, quinone is the basic moiety of this compound, which is also a product of the shikimate pathway. *OsCM* is not concerned with the synthesis of the second unit of the compound, but it is necessary to describe its synthesis briefly. It has been reported that oxadecalin is the derivative of glycosyl cyanide by cycloaddition reaction with ketone and reductive alkylation with cyclopropylketone. It can be summarized that *OsCM* has a vital role in the shikimate pathway due to upregulation of both Tyr and Phe, which is the ultimate source of catechol and the farnesyl moiety in the synthesis of both cq-9 and epi-cochlioquinone, respectively.

Cochlioquinone has already been reported to cause toxic reactions in various cancer cells [60,61]. In particular, Zhou et al. (2015) [62] identified that cochlioquinone caused cytotoxicity in the A549 cell line and SK-OA-3 cell line, which are cells that mainly cause cancer in humans. We succeeded in extracting cq-9 from rice for the first time and inoculated into the sepsis-inducing model mice. Moreover, the survival rate was increased by 60% in sepsis-induced model. It has also been reported that cq-9 has antimicrobial activity against *Phythium graminicola* [63], which causes severe damage in rice seedlings [64].

Rice extract or cq-9 induced significant reduction of U46619 -mediated contraction in endothelium-intact compared with endothelium-denuded rat aorta rings. Furthermore, rice extract or cq-9 induced a dose-dependent vasodilation in endothelium-intact rat aortic rings, which was attenuated by L-NAME, a nitric oxide synthase blocker. These findings suggest that endothelium-dependent relaxation induced by rice extract or cq-9 leads to activation of eNOS, resulting in production of NO in endothelial cells [65]. However, pretreatment with a K^+^ channel blocker, apamin or TAE, did not affect endothelium-dependent vasorelaxation induced by rice extract or cq-9, indicating that endothelium-dependent vasorelaxation by rice extract or cq-9, does not mediate the K^+^ channel pathway. Furthermore, this vasodilation proved that cq-9 was higher than that of rice extract. Rice extract or cq-9 extract inhibited collagen-, ADP- and thrombin-induced platelet aggregation in a dose-dependent manner. Furthermore, this collagen-induced platelet aggregation proved that cq-9 was higher than that of rice extract.

In this research, 120 CNDH lines were analyzed to QTL mapping associated with WBPH resistance. QTLs were analyzed using the phenotype of the 120 CNDH lines and HPLC analysis data, and finally *OsCM* was screened. *OsCM* is an important regulatory enzyme in synthesizing cq-9, and *OsCM* transgenic rice is highly resistant to WBPH. When cq-9 was treated in CLP-surgery mice, the survival was increased by 60%. Furthermore, the aorta of mice treated with cq-9 had an effective vasodilation response, and significantly reduced the aggregation of rat platelets induced by collagen treatment. The results of this research show that cq-9 produced by plants can be effective in treating animal diseases and can be effectively used to study the relationship between Plant-Insect-Human in the future.

## 5. Conclusions

In this research, the effects of cq-9, a substance produced by the *OsCM* gene mapped in rice, on survival rate and vascular relaxation in CLP-surgery mice were analyzed. For screening *OsCM*, both phenotype and secondary metabolite data were used, and these data were finally detected in the region to which they are commonly mapped. When *OsCM* was overexpressed, symptoms caused by WBPH hardly occurred. WBPH induces plant disease by mediating the virus to plants. Therefore, when cq-9, a substance that induces resistance to plant viruses, was applied, the survival rate and vasorelaxation of CLP-surgery mice were analyzed positively. Furthermore, cq-9 increases the survival rate of CLP-surgery mice by 60%. Cq-9 reduced rat platelets aggregation. In this study, cq-9, a pest-resistant substance produced by plants, suggests the possibility of application in the treatment of sepsis caused by viruses and vasoconstriction in animals. Therefore, cq-9 can be effectively used to elucidate the relationship between plant and insect or study their interaction.

## Data Availability

Data are contained within the article and Supplementary Materials.

## Supplementary Materials

The following are available online at https://www.mdpi.com/article/10.3390/antiox10111691/s1. Table S1. QTLs associated with resistance to WBPH in Cheongcheong/Nagdong double haploid lines for 5 years, Table S2. Eight candidate genes between the RM23230-RM3659 and their ORFs, which include various proteins related to WBPH, Figure S1. Extraction of substances related to cq-9. Figure S2. Schematic representation of the shikimate pathway in plants.

## Abbreviations

BSA	bovine serum albumin
CLP	caecal ligation puncture
CM	chorismate mutase
CNDH	Cheongcheong/Nagdong double haploid
HMGB1	high mobility group protein 1
HPLC	high-performance liquid chromatography
HUVECs	human umbilical vein endothelial cells
LOD	logarithm of odds
LPS	lipopolysaccharide
2D-PAGE	two-dimensional polyacrylamide gel electrophoresis
QTL	quantitative trait loci
RFLP	restriction fragment length polymorphism
SM	secondary metabolite
TN1	Taichung Native 1
TLC	thin-layer chromatography
WBPH	whitebacked planthopper
