# Supplementary material for: The Quantitative Trait Loci Mapping of Rice Plant and the Components of Its Extract Confirmed the Anti-Inflammatory and Platelet Aggregation Effects In Vitro and In Vivo
Source: Antioxidants (Basel). 2021 Oct 26;10(11):1691. doi: 10.3390/antiox10111691 (PMC8615199; doi:10.3390/antiox10111691)
Supplement: Supplementary file 1 [file antioxidants-10-01691-s001.zip › antioxidants-1417384-supplementary.pdf]

antioxidants

MDPI

Supplementary Materials: The Quantitative Trait Loci Mapping of Rice Plant and the Components of Its Extract Confirmed the Anti-Inflammatory and Platelet Aggregation Effects In Vitro and In Vivo

Jae-Ryoung Park [1,2,†], Rahmatullah Jan [1,2,†], Seul-Gi Park [3,†], Tri Handoyo [4,†], Gang-Seob Lee [5,†], Sopheap Yun [6], Yoon-Hee Jang [1,2], Xiao-Xuan Du [2,5], Taeho Lee [7], Yong-Sham Kwon [8], Doh Hoon Kim [8], Young-Mi Seok [9], Jong-Sup Bae [7] and Kyung-Min Kim [1,2,*]

[1] Division of Plant Biosciences, School of Applied Biosciences, College of Agriculture & Life Science, Kyungpook National University, 80 Dahak-ro, Buk-gu, Daegu 41566, Korea; icd92@knu.ac.kr (J.-R.P.); rahmat2021@knu.ac.kr (R.J.); uni@knu.ac.kr (Y.-H.J.)

[2] Costal Agriculture Research Institute, Kyungpook National University, 80 Dahak-ro, Buk-gu, Daegu 41566, Korea; duxiaoxuan@korea.kr

[3] National Institute of Crop Science, Rural Development Administration, Jeonju 54874, Korea; ahsia1004@korea.com

[4] Department of Agronomy, Faculty of Agriculture, Jember University, Jl. Kalimantan 37, Jember 68121, Indonesia; trihandoyo.faperta@unej.ac

[5] Biosafety Division, National Academy of Agricultural Science, Rural Development Administration, Jeonju 54874, Korea; kangslee@korea.kr

[6] Graduate School of Science, Royal University of Phnom Penh, Sangkat Teuk Laak 1, Russian Federation Boulevard, Toul Kork, Phnom Penh 12101, Cambodia; yun.sopheap@rupp.edu.kr

[7] College of Pharmacy, Research Institute of Pharmaceutical Sciences, Kyungpook National University, 80 Dahak-ro, Buk-gu, Daegu 41566, Korea; tlee@knu.ac.kr (T.L.); baejs@knu.ac.kr (J.-S.B.)

[8] Department of Genetic Engineering, College of Natural Resources and Life Science, Dong-A University, Busan 49315, Korea; kkmkim@hanmail.net (Y.-S.K.); dhkim@dau.ac.kr (D.H.K.)

[9] Department of Korean Medicine Development, National Institute for Korean Medicine Development, 94, Hwarang-ro, Gyeongsangbuk-do, Gyeongsan-si 38540, Korea; imaria@nikom.or.kr

* Correspondence: kkm@knu.ac.kr; Tel.: +82-53-950-5711

† These authors contributed equally to this work.

Citation: Park, J.-R.; Jan, R.; Park, S.-G.; Handoyo, T.; Lee, G.-S.; Yun, S.; Jang, Y.-H.; Du, X.-X.; Lee, T.; Kwon, Y.-S.; et al. The Quantitative Trait Loci Mapping of Rice Plant and the Components of Its Extract Confirmed the Anti-Inflammatory and Platelet Aggregation Effects In Vitro and *In Vivo*. *Antioxidants* **2021**, *10*, 1691. https://doi.org/10.3390/antiox10111691

Academic Editor: Naphtali Savion

Received: 27 September 2021
Accepted: 23 October 2021
Published: 26 October 2021

Academic Editor: Naphtali Savion

Publisher's Note: MDPI stays neutral with regard to jurisdictional claims in published maps and institutional affiliations.

Copyright: © 2021 by the authors. Submitted for possible open access publication under the terms and conditions of the Creative Commons Attribution (CC BY) license (http://creativecommons.org/licenses/by/4.0/).

Table S1. QTLs associated with resistance to WBPH in Cheongcheong/Nagdong double haploid lines for 5 years.

Year	QTLs	Chr.	Interval Markers [z]	LOD	Additive effect [y]	R^{2x}	Increasing effects[w]
2016	qwbph2	2	RM5619-RM424	2.5	1.3	0.10	Cheongcheong
	qwbph4	4	RM273-RM16467	4.7	1.8	0.18	Cheongcheong
	qwbph6	6	RM50 -RM1163	6.1	2.1	0.29	Cheongcheong
	qwbph7	7	RM21972-RM6776	4.8	1.7	0.18	Cheongcheong
	qwbph8	8	RM3689-RM23314	5.2	1.5	0.18	Cheongcheong
2017	qwbph4-1	4	RM127-RM17502	2.6	1.4	0.16	Cheongcheong
	qwbph6-1	6	RM50-RM1163	4.3	1.7	0.23	Cheongcheong
	qwbph6-2	6	RM20196-RM20096	2.7	1.1	0.11	Cheongcheong
2018	qwbph4-2	4	RM280-RM6909	3.5	1.1	0.30	Cheongcheong
	qwbph7-1	7	RM248-RM1134	3.0	1.2	0.30	Cheongcheong
	qwbph8-1	8	RM23230-RM3689	2.5	-1.5	0.30	Nagdong
	qwbph8-2	8	RM17699-RM264	3.3	0.7	0.30	Cheongcheong
	qwbph12	12	RM1226-RM12	2.7	1.1	0.40	Cheongcheong
2019	qwbph1	1	RM3482-RM11966	4.0	-0.3	0.30	Nagdong
	qwbph1-1	1	RM3709-RM11694	3.5	0.3	0.30	Cheongcheong
	qwbph1-2	1	RM11694-M11669	3.3	0.5	0.30	Cheongcheong
	qwbph8-3	8	RM17699-RM264	3.3	0.7	0.30	Cheongcheong
2020	qwbph4-3	4	RM280-RM6909	3.5	1.1	0.30	Cheongcheong
	qwbph6-3	6	RM248-RM1134	3.0	1.2	0.30	Cheongcheong
	qwbph8-4	8	RM23230-RM3689	2.5	-1.5	0.30	Nagdong
	qwbph12-1	12	RM1226-RM12	2.7	1.1	0.40	Cheongcheong

[z] Interval markers are those within the significance threshold on each border of the QTL range.

[y] The proportion of evaluated phenotype variation attributable to a particular QTL was estimated by the coefficient of determination (R^2).

[x] Positive values of the additive effect indicate that alleles from Cheongcheong are in the direction of increasing the traits.

[w] Increase allele is the source of the allele causing an increase in the measured trait.

Table S2. Eight candidate genes between the RM23230-RM3659 and their ORFs, which include various proteins related to WBPH.

No.	Chr.	Gene locus	Gene	Description	Function
1	8	Os08g0453700	OsNox6, OsrbohE	*Arabidopsis thaliana* respiratory burst oxidase protein F; NAD(P)H oxidase	Plant defence responses (direct killing)
2	8	Os08g0441500	OS-CCR, OsCCR, CCR, CCR20	Cinnamoyl-CoA reductase	Response to infection with pathogenic bacteria
3	8	Os08g0441600	OsCM	Similar to chorismate mutase CM2 (EC 5.4.99.5) (chorimate mutase). (Os08t0441600-01)	Synthesises prephenate
4	8	Os08g0459600	OsOPR7, OsOPR13,	12-Oxophytodienoate reductase (EC:1.3.1.42), jasmonic acid biosynthesis (Os08t0459600-01)	Phytodienoic acid reductase 7, 12-oxophytodienoate reductase7, OPDA reductase 7
5	8	Os08g0458600	OsRR33, Rra19, OsRRA19	Signal transduction response regulator, receiver region domain containing protein	Response regulator 33, A-type response regulator 19, A-type RR 19
6	8	Os08g0460000	OsGLP1	Germin-like protein 1 precursor	Plant defence
7	8	Os08g0472000	OsbZIP66, Osbzip66	bZIP transcription factor, abscisic acid-regulated transcription	Regulator of plant defence and development
8	8	Os08g0474000	OsDERF3,	AP2 domain containing protein RAP2.6	Regulator of plant defence

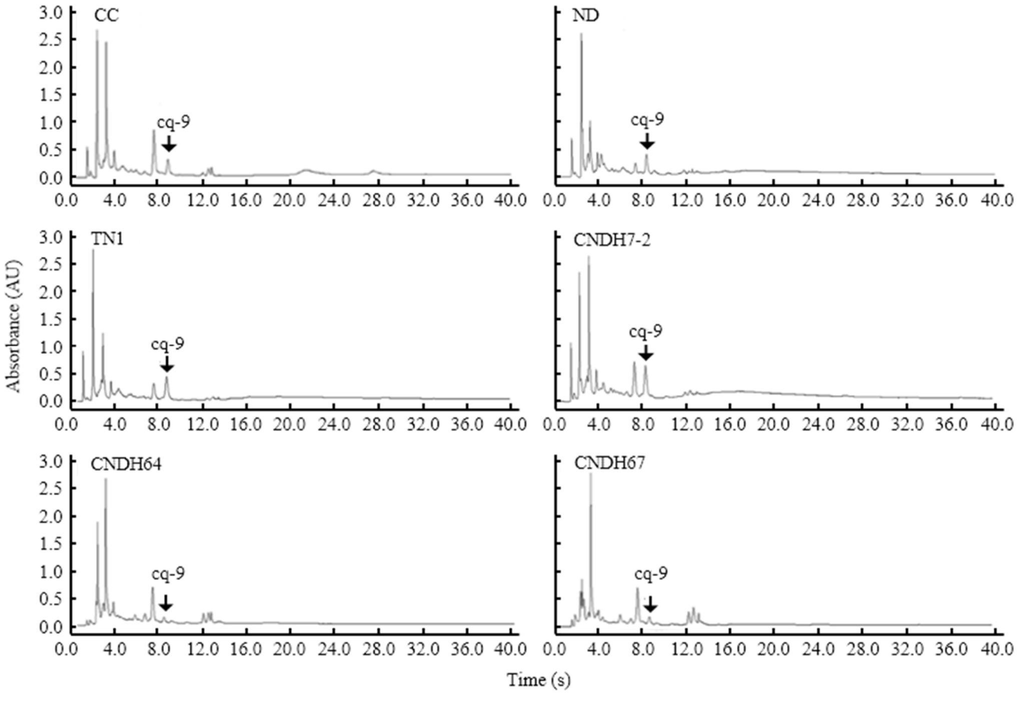

Figure S1. Extraction of substances related to cq-9. Typical HPLC chromatogram of cq-9. Typical chromatogram of cq-9 in the extract of rice samples determined by HPLC at 254 nm. Cq-9 is made in high concentrations in resistant populations after WBPH inoculation. However, in a susceptible

population, it is made at a very low level. And a moderative population is created with a concentration intermediate between susceptible and resistance. CNDH64 and CNDH67 are susceptible populations, and Cheongcheong and Nagdong are moderative cultivars. TN1 and CNDH7-2 are resistance populations. CC; Cheongcheong, ND; Nagdong.

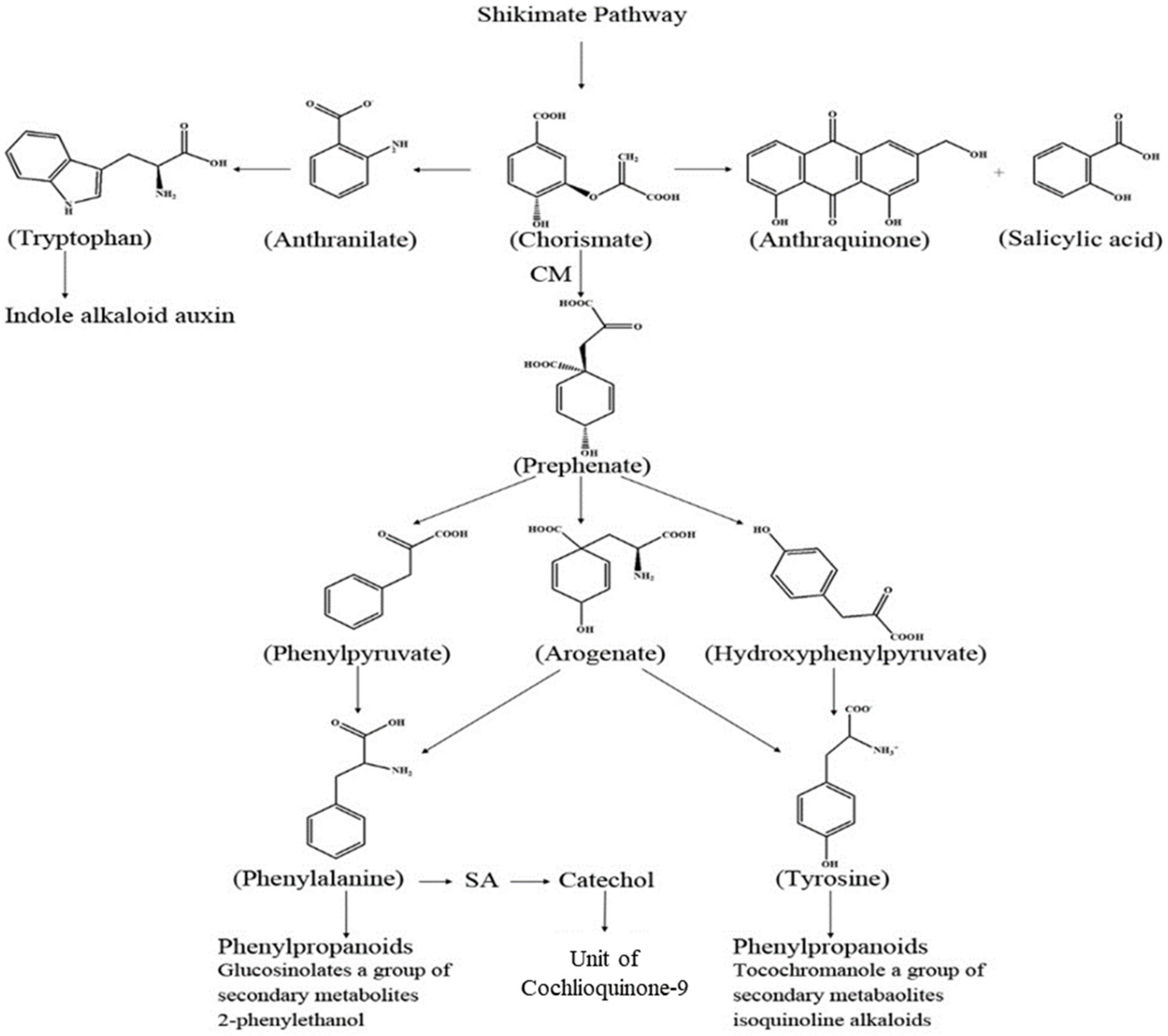

Figure S2. Schematic representation of the shikimate pathway in plants. This is the main pathway of biosynthesis of aromatic amino acids (phenylalanine, tyrosine and tryptophan), which are the precursor of various volatile compound and secondary metabolites. In this pathway, the key step is the conversion of chorismate into prephenate, which is the main precursor of aromatic amino acids. Chorismate mutase enzyme is involved in the conversion of chorismate into prephenate. Phenylalanine is the precursor of the phenylpropanoid pathway, which is the intermediate of a high diversity of metabolites, including flavonoids and anthocyanins. The shikimate pathway produces salicylic acid (SA) in two ways; the *iso*-chorismate synthase enzyme converts anthraquinone into SA in the initial steps, while, in the last steps, phenylalanine undergoes a series of reactions and produces SA. SA is the ultimate source of catechol goes under various reaction, which is a key unit of cq-9.
